# Assisted oocyte activation re-envisioned: a more physiological approach

**DOI:** 10.1007/s10815-026-03921-2

**Published:** 2026-06-16

**Authors:** Philip Xie, Stephanie Cheung, Lily Ng, Zev Rosenwaks, Gianpiero D Palermo

**Affiliations:** The Ronald O. Perelman and Claudia Cohen Center for Reproductive Medicine, Weill Cornell Medicine, New York, United States

**Keywords:** ICSI, Fertilization failure, Assisted oocyte activation, PLCζ

## Abstract

**Purpose:**

To determine the gamete-specific etiology of severe or total fertilization failure after intracytoplasmic sperm injection (ICSI) and to evaluate the efficacy of individualized assisted oocyte activation (AOA) strategies guided by sperm phospholipase C-zeta (PLCζ) content.

**Methods:**

A pilot topographic analysis on PLCζ content in ejaculated, epididymal, and testicular spermatozoa was performed to better understand the fertilization capability of spermatozoa at different maturational stages. The prospective case series included 135 couples with complete or severe fertilization failure following ICSI, who were screened for PLCζ content and allocated as having oocyte- or sperm-related deficiency based on PLCζ assay results. According to gamete-specific deficits, cycles were treated with AOA, that is, ionophore-only, rhPLCζ-only, or combined treatment. Embryological and clinical outcomes of AOA cycles were compared with those of historical non-AOA ICSI cycles.

**Results:**

PLCζ content varied according to the maturity and origin of spermatozoa in the pilot study. Compared with historical cycles, AOA using ionophore and rhPLCζ each rescued fertilization and improved pregnancy outcomes with comparable efficiency. Notably, dual AOA (ionophore + rhPLCζ) yielded the greatest improvements in fertilization (24.1% vs. 73.2%, *P* < 0.0001), implantation (6.5% vs. 31.7%, *P* < 0.01), and ongoing pregnancy rates (0% vs. 63.3%, *P* < 0.0001), even in cycles using testicular spermatozoa.

**Conclusions:**

The introduction of recombinant PLCζ enabled specific correction of gamete-specific fertilization failure. Dual AOA (ionophore + rhPLCζ) was particularly effective in cases of compounded etiology and with immature spermatozoa, supporting a shift from empiric to individualized AOA strategies.

**Supplementary Information:**

The online version contains supplementary material available at https://doi.org/10.1007/s10815-026-03921-2.

## Introduction

The introduction of in vitro fertilization (IVF) in the late 1970 s transformed the treatment of infertility by ensuring direct exposure of oocytes to spermatozoa; however, conventional in vitro insemination does not resolve situations in which spermatozoa are limited in number, demonstrating poor kinetic profiles, or are unable to penetrate the zona pellucida due to morphological anomalies and may result in severe or total fertilization failure in at least 10% of cases [1]. In contrast, intracytoplasmic sperm injection (ICSI) directly introduces spermatozoa into the cytoplasm of oocytes regardless of the morphology and motility of the male gametes [2, 3]. ICSI is an effective approach to overcome failed fertilization observed with conventional insemination [4] and has become a popular IVF technique, accounting for 57.1% of all assisted reproductive technique cycles [5]. Nonetheless, despite more direct insemination, total fertilization failure still occurs in 1–3% of ICSI cycles [6], almost exclusively related to the male gamete [7, 8]. With the widespread adoption of ART with ICSI, total or severe fertilization failure may affect approximately 200,000 couples annually.

At fertilization, the sperm cell, once inside the oocyte, releases its labile cytosolic factor phospholipase C-zeta (PLCζ), which triggers the intracellular calcium oscillations required for oocyte activation [9, 10]. In patients lacking functional PLCζ due to mutations [11], organelle abnormalities such as globozoospermia [12], or in the case of testicular spermatozoa, which are known to have lower fertilization potential, fertilization failure may occur. Diagnostic evaluation of PLCζ deficiency includes direct observation of PLCζ localization using immunofluorescence [8]. Additional biological assays such as mouse oocyte activation test (MOAT) directly assess the fertilization competence of spermatozoa of the patient [13], while functional assays such as mouse oocyte calcium analysis (MOCA) and human oocyte calcium analysis (HOCA) investigate calcium oscillation pattern elicited by the spermatozoa within mouse or human oocytes, respectively [14, 15]. Genetic testing is also performed to identify gene mutation responsible for activation deficiency [8, 11, 16]. Assisted oocyte activation (AOA) has become a reliable treatment strategy for patients with recurrent fertilization failure following ICSI, aiming to induce a cytosolic Ca^2^⁺ surge that mimics the oscillatory pattern observed during physiological fertilization [8, 13]. Among available AOA methods, including mechanical activation, electroactivation, or chemicals such as strontium chloride, puromycin, or cycloheximide [17], the most popular AOA protocol uses calcium ionophores. This approach is widely adopted because of its simplicity and reproducibility and is supported by clinical follow-up studies demonstrating no adverse effects on neonatal or offspring developmental outcomes [18, 19].

Despite its overall success, calcium ionophore induces a single, unsynchronized calcium signal, which may not always be adequate to sustain the subsequent calcium oscillations as revealed in MOCA test, revealing a sudden surge of intracellular calcium level in murine oocytes [20]. Consequently, it is not always reliable in rescuing fertilization or supporting subsequent embryo development, reflecting its inability to fully compensate for intrinsic PLCζ function [21, 22]. In cases of failed fertilization, an oocyte-related factor may also be involved, such as a genetic defect disrupting the molecular pathway of oocyte activation [7, 23] or subtle cytoplasmic dysmaturity due to suboptimal timing of the superovulation process, which can be simply addressed by modifying the stimulation protocol [8, 24, 25]. However, the mechanism underlying oocyte-related fertilization failure has not been fully elucidated, although some observations and clinical reports suggest that AOA may be beneficial in such cases [6, 21, 26].

A more physiological approach to oocyte activation is currently needed [27]. Human PLCζ is a potent trigger of oocyte activation [28]. Early attempts focused on the use of sperm extracts containing this soluble factor to induce oocyte activation and support fertilization [15, 29], while more recent approaches using genetic engineering have enabled the production of recombinant human PLCζ (rhPLCζ), showing preliminary success in enhancing oocyte activation in mammalian oocytes [27, 30].

This study aimed to determine the contribution of each gamete to low or absent fertilization. Accordingly, all couples included in the study with a history of poor fertilization underwent screening for PLCζ. Moreover, to better understand the fertilization capability of spermatozoa at different maturational stages and retrieved from different regions of the male genital tract, we assessed the topographical distribution of PLCζ in a pilot study. Based on PLCζ results, couples were classified as having oocyte- or sperm-related fertilization failure. For suspected oocyte contribution, calcium ionophores were used to specifically address fertilization deficiency. In contrast, for those lacking PLCζ, rhPLCζ was used to replace the absent labile sperm-derived oocyte-activating factor. In cases in which the etiology was unclear or concurrently related to oocytes and spermatozoa, a combined AOA approach was applied. In addition, the recombinant protein was tested in spermatozoa retrieved directly from the seminiferous tubules. To evaluate the developmental competence of embryos derived from gametes exposed to AOA, fertilization and clinical outcomes were assessed following ionophore-only, rhPLCζ-only, and dual AOA protocols, each addressing distinctive gamete deficiencies.

## Materials and methods

### Inclusion criteria and study design

Over a 25-year period, this prospective case series included couples with a history of complete fertilization failure or consistently low fertilization rates (< 30% two-pronuclei [2PN] rate) following conventional ICSI, who subsequently underwent treatment cycles with AOA within 12 months with respect to the history cycle. The study was approved by the Institutional Review Boards (IRB# 0712009553 and 1006011085). All patients included in this study were counseled extensively and individually regarding the benefits and risks involving oocyte activation treatment, including the detail about the specific addition of the activating agent at the time of ICSI. Consenting patients acknowledged that the technique is novel and involves currently unknown aspects that may pose additional risks to the gametes and developing embryos.

To understand the contribution of specific gametes, PLCζ content was assessed. Based on our experience with the topographic retrieval of spermatozoa and their DNA integrity [31], we evaluated PLCζ in ejaculated spermatozoa and those retrieved from the proximal male genital tract, including the epididymis and testis. The diagnosis of sperm- or oocyte-related fertilization failure was determined based on sperm PLCζ content, using a ≥ 30% normal threshold. If the PLCζ assay yielded normal levels, couples were classified as having oocyte-related fertilization failure. Conversely, low PLCζ levels indicated sperm-related fertilization failure and were corroborated by genetic assessment to identify specific gene mutations responsible for fertilization failure.

Different AOA protocols were offered depending on the diagnosis. For oocyte-related fertilization failure, AOA using calcium ionophore only was applied, whereas for sperm-factor cases, rhPLCζ alone was used as the AOA agent. In cases with suspected combined or unexplained etiology, such as borderline PLCζ levels, or in cycles using testicular spermatozoa, a dual AOA protocol, combining rhPLCζ coinjection during ICSI and post-ICSI exposure to calcium ionophore, was employed.

Cycles were categorized according to the gamete source responsible for fertilization failure and treated with the corresponding AOA modalities. Embryological and clinical outcomes of AOA treatment cycles were compared with those of the patients’ historical non-AOA cycles. In addition, couples undergoing ICSI with testicular spermatozoa were treated with dual AOA because of the consistently low PLCζ content observed in these samples. A subgroup analysis was performed for cycles utilizing testicular spermatozoa.

### Semen analysis and preparation

Ejaculated semen samples were obtained by masturbation after 2–5 days of abstinence. The semen specimens were allowed to liquefy in 37 °C and then analyzed according to the World Health Organization sixth edition manual [32]. Samples were processed using standard density gradient centrifugation and diluted to a concentration of 1–2 million sperm/mL for ICSI.

Testicular sperm extraction was performed using standard surgical techniques in patients requiring surgical sperm retrieval. Retrieved testicular tissue was mechanically dispersed, and viable spermatozoa were identified in an ICSI dish under an inverted microscope [33]. Motile or twitching spermatozoa were selected when available. Both fresh and cryopreserved testicular spermatozoa were used according to clinical availability.

### PLCζ assessment

**Fig. 1:**
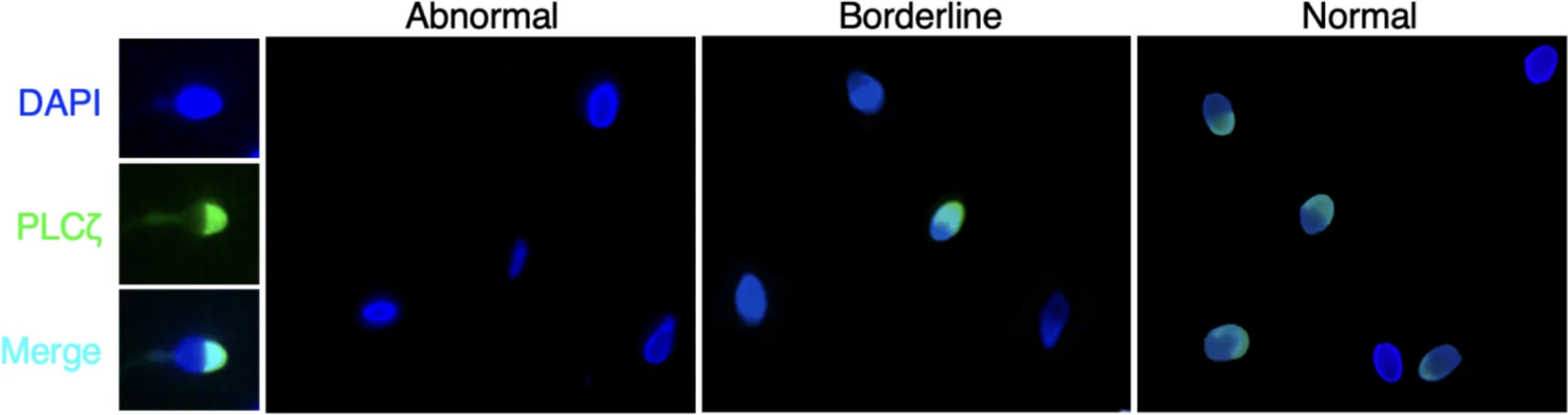
Visualization of PLCζ expression; sperm DNA is visualized by DAPI staining in blue; PLCζ is stained with green fluorescence

Sperm PLCζ assessment was carried out with minor modifications to a previously described protocol [8]. An aliquot of semen (5–10 µL) was smeared onto glass slides. Spermatozoa were fixed in 4% paraformaldehyde and dried overnight. Slides were permeabilized with 0.5% Triton X-100 at room temperature for 10 min and treated with Pierce Protein-Free Blocking Buffer (37,572, Thermo Fisher Scientific). Slides were then incubated overnight at 4 °C with a polyclonal anti-human PLCζ primary antibody (PA5-50,476, Thermo Fisher Scientific). Specimens were labeled with an anti-rabbit immunoglobulin G secondary antibody and concurrently counterstained with 4ʹ,6-diamidino-2-phenylindole (5 μg/mL) to identify sperm nuclei. Imaging was performed at × 100 magnification using a fluorescence microscope equipped with fluorescein isothiocyanate filters. PLCζ immunofluorescence localization within the acrosomal, equatorial, and post-acrosomal regions of the sperm head was evaluated in a minimum of 200 spermatozoa per specimen, and the proportion of PLCζ-positive cells was recorded (Fig. 1). A threshold of ≥ 30% PLCζ-positive spermatozoa was considered normal based on our laboratory reference range established from a large cohort of patients with extremely poor fertilization [8].

### Sperm genome analysis

To identify gene mutations associated with the oocyte-activating capacity of spermatozoa, DNA sequencing was performed as described previously [34]. Genomic DNA was extracted and amplified using a commercial kit (Repli-G Single Cell; Qiagen, Hilden, Germany). Sequencing was performed at an external facility (Genewiz, Azenta Life Science). Copy number variation analysis classified genes as duplicated when the read depth exceeded 1.5-fold the median read depth, or the number of unique reads, of the control group across > 70% of exons, and as deleted when the read depth was < 0.5-fold the corresponding control median.

### rhPLCζ preparation and validation

Custom-made rhPLCζ was designed and purified by a third-party company (GenScript). The 64.5-kDa recombinant protein was synthesized in BL21 Star™ (DE3) *E. coli* and purified using nickel-affinity chromatography followed by Superdex 200 size-exclusion chromatography. The purified rhPLCζ was suspended in buffer containing 50 mM Tris–HCl, 150 mM NaCl, 1 M L-arginine, and 10% glycerol, at a final concentration of 0.97 mg/mL.

Before application to human gametes, the protein was tested in mouse oocytes. Murine oocytes were obtained from the oviducts of superovulated B6D2F1 adult female mice. A small volume (approximately 0.4 pL) of rhPLCζ suspension was injected into the ooplasm using a piezo-actuated microinjector. Unmanipulated and sham-injected oocytes served as negative controls, while calcium ionophore-treated oocytes were used as positive controls. Oocytes were then placed in a time-lapse incubator to monitor PN formation and assess oocyte activation.

### Controlled ovarian hyperstimulation and oocyte retrieval

The choice of ovarian stimulation protocol was determined based on patient age, body mass index, antral follicle count, serum anti-Müllerian hormone levels, and prior response to ovarian stimulation [35]. Controlled ovarian hyperstimulation was achieved by daily administration of gonadotropins (Follistim, Merck; Gonal-F, EMD-Serono; Menopur, Ferring Pharmaceuticals). Pituitary suppression was achieved using either a gonadotropin-releasing hormone agonist (leuprolide acetate, Abbott Laboratories) or antagonist (ganirelix acetate, Merck; or cetrorelix, EMD-Serono). In selected patients, oral contraceptive pretreatment (Ortho-Novum; Janssen Pharmaceuticals) was used to promote follicular synchronization before gonadotropin administration. Final oocyte maturation was triggered with human chorionic gonadotropin (hCG, Ovidrel, EMD-Serono) when at least two leading follicles reached a diameter of ≥ 17 mm. Transvaginal ultrasound-guided oocyte retrieval was performed under conscious sedation 35–37 h after the hCG trigger.

Retrieved cumulus-oocyte complexes (COCs) were incubated for a minimum of 2.5 h. The COCs were exposed briefly (≤ 30–60 s) to 40 IU/mL hyaluronidase (Cumulase, Halozyme Therapeutics) to facilitate loosening of the extracellular matrix. Mechanical denudation was performed on COCs immediately after, using a series of calibrated stripper pipettes (Origio), beginning with a larger bore (200-μm inner diameter) to remove the bulk of the cumulus mass, and subsequently transitioning to smaller-diameter pipettes (e.g., 170–150 μm) to completely remove tightly adherent cumulus cells. Complete removal of residual corona radiata was required to ensure unobstructed visualization of the oocyte and polar body for assessment of nuclear maturity and to facilitate appropriate positioning of the holding and injection pipettes during ICSI. Denuded oocytes were washed twice in culture medium and examined under an inverted microscope to assess oocyte morphology and nuclear maturity. Oocytes exhibiting a clear first polar body were classified as metaphase II (MII) and deemed suitable for ICSI.

### ICSI with conventional AOA, rhPLCζ-AOA, and dual-agent AOA

For oocyte-factor cases, AOA using ionophore only was employed based on previous study [8]. Briefly, spermatozoa were briefly exposed to 50 µM calcium ionophore (A23187, Sigma Aldrich) for 5 min before ICSI. During ICSI, individual spermatozoa displaying erratic motility were selected, aspirated, and immobilized in separate drops of polyvinylpyrrolidone. Approximately 0.4 pL of calcium ionophore medium was then aspirated into the micropipette alongside the spermatozoon and coinjected into the oocyte. Following ICSI, oocytes were further exposed to 50 μM calcium ionophore for 10 min at 37 °C, after which they were thoroughly rinsed in culture medium and transferred to culture medium.

In cases utilizing rhPLCζ-AOA, spermatozoa were injected with approximately 0.4 pL of 0.97 mg/mL rhPLCζ (approximately 3.6 million molecules) into the oocyte during ICSI without additional exposure.

In treatment cycles where a combined AOA modality was employed, 0.4 pL of rhPLCζ was coinjected at ICSI. Post-ICSI, oocytes were then exposed to 50 mM calcium ionophore for 10 min and thoroughly washed before culture.

### Assessment of fertilization, embryo replacement, and pregnancy follow-up

Successful oocyte activation and fertilization were assessed 16–18 h after ICSI using an inverted microscope or a time-lapse incubator. An oocyte was considered activated when at least one pronucleus was visible. Successful fertilization was defined as the extrusion of the second polar body in the presence of two distinct pronuclei.

Fresh embryo transfer was performed on day 3 or 5 after oocyte retrieval, whereas frozen embryo transfer of blastocysts was performed on day 5 after ovulation in natural or programmed cycles. Luteal phase support consisted of daily intramuscular progesterone supplementation (50 mg), initiated 24 h after oocyte retrieval. The implantation rate was defined as the number of gestational sacs divided by the number of transferred embryos. Clinical pregnancy was defined as the presence of at least one fetal heartbeat recorded by transvaginal ultrasonography at 7 weeks of gestation. Delivery and neonatal outcome data were obtained from obstetrician and pediatrician records.

### Statistical analysis

The *t*-test was used to compare semen parameters and PLCζ content. Chi-square and Fisher’s exact tests were used to compare outcomes between historical ICSI cycles and subsequent cycles employing AOA. Statistical significance was defined as a two-sided *P* value of < 0.05.

## Results

### Fertilization capability according to sperm maturity and origin

**Fig. 2:**
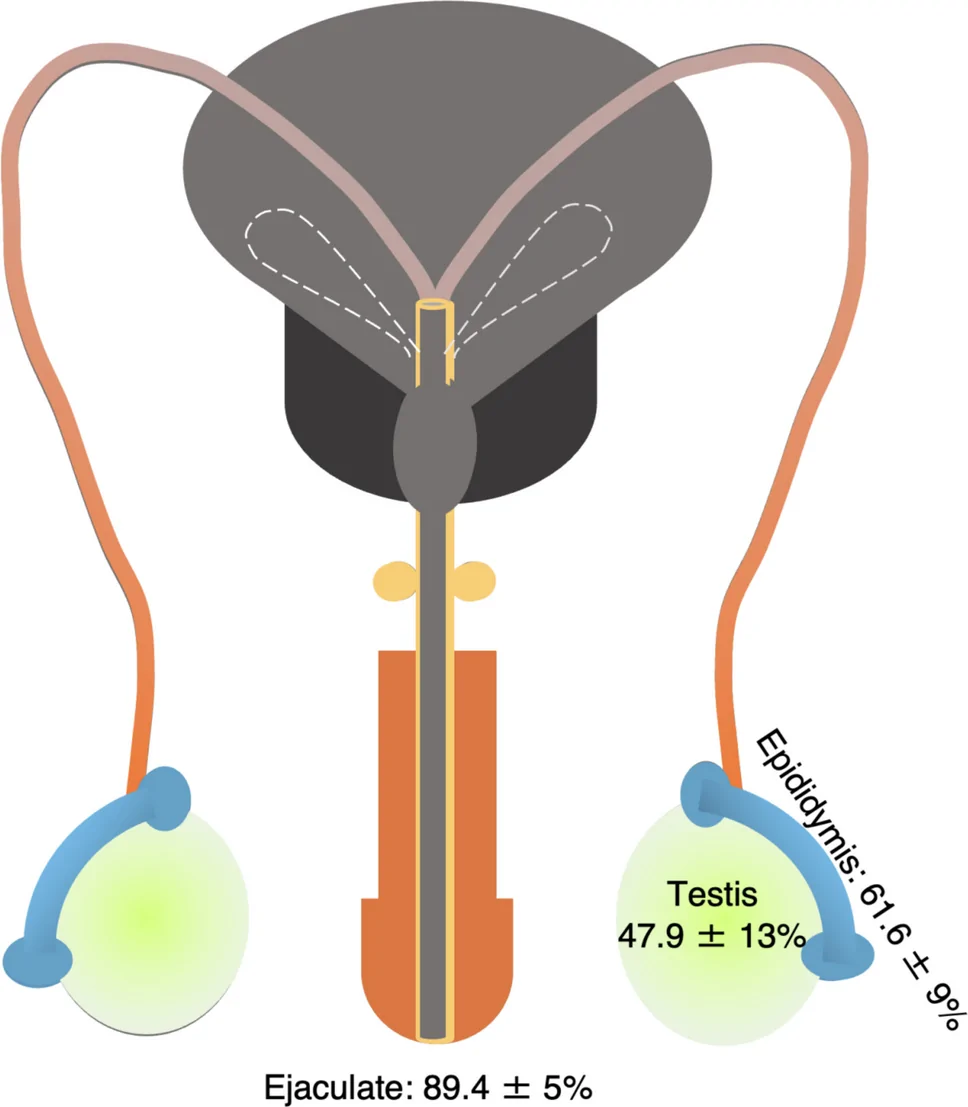
Topographical expression of PLCζ within the male genital tract, displaying a reduction of PLCζ expression as spermatozoa are retrieved more proximally

PLCζ assessment was carried out in a pilot study (*n* = 6) of men with normal spermatogenesis who underwent surgical sperm retrieval due to high DNA fragmentation in their ejaculate and consented to have PLCζ assessment performed on their ejaculated, epididymal, and testicular spermatozoa. Testicular spermatozoa displayed the lowest level of PLCζ (Fig. 2).

### PLCZ level and semen characteristics

A total of 135 couples (maternal age 36.3 ± 5; paternal age 36.9 ± 5) with a history of poor fertilization rate (mean 2PN rate 19.6%) were included in this study. Semen analysis revealed a volume of 2.4 ± 2 mL, concentration of 42.7 ± 40 × 10^6^/mL, motility of 33.9 ± 21%, and normal morphology of 2.6 ± 2%, with PLCζ of 31.3 ± 26%. Semen parameters and PLCζ levels specific to each patient category are presented in Table 1.

**Table 1:** Patient semen parameters and PLCζ content in couples classified according to a specific gamete contribution to fertilization failure

	Oocyte factor	Sperm factor	Low/borderline PLCζ
**Couples (*n*)**	77	20	38
Female age	36.2 ± 6	36.6 ± 6	36.4 ± 5
Male age	38.1 ± 8	39.7 ± 8	38.3 ± 7
**Semen parameters**			
Volume (mL ± SD)	2.5 ± 2	3.3 ± 3	2.2 ± 1
Concentration (10^6^/mL ± SD)	43.3 ± 44	42.1 ± 35	39.4 ± 32
Motility (% ± SD)	35.8 ± 22	30.8 ± 23	31.9 ± 17
Normal morphology (% ± SD)	3.0 ± 2^*^	2.0 ± 1^*^	2.4 ± 2
PLCζ positivity (% ± SD)	68.2 ± 17	5.8 ± 3	32.6 ± 14

^*^P < 0.05 by unpaired *t*-test

Next-generation sequencing assessment of spermatozoa identified mutations in genes essential for sperm-egg binding (ADAM2 and *ADAM15*), meiotic differentiation of spermatocytes (*DPY19L2, PICK1, PIWIL1,* and *TDRD6*), calcium signaling (*CACNG7, CATSPER1, CATSPERG, MCTP2,* and *CGREF1*), and other genes involved in cell signaling during fertilization (*SRCIN1, KCNC3*). Mutations in *PLCZ1*, responsible for eliciting calcium oscillations and oocyte activation during fertilization, were also identified.

### Efficiency of rhPLCζ

In preliminary experiments using murine oocytes, a total of 160 MII oocytes were allocated for sham injection (*n* = 20), unmanipulated (*n* = 20), calcium ionophore exposure (*n* = 20), and rhPLCζ injection (*n* = 100). Sham-injected and unmanipulated oocytes did not display polar body extrusion or PN formation after 6 h of monitoring in the time-lapse incubator. Calcium ionophore-treated oocytes were 80.0% (16/20) activated, as confirmed by 1PN formation. Oocytes injected with rhPLCζ activated at 76.0% (76/100), comparable to the conventional ionophore method.

### Ionophore-only AOA boosts fertilization in oocyte-related activation deficiency

In patients treated with ionophore-only AOA to address suspected oocyte-related issues, the fertilization rate significantly improved compared to historical cycles (52.5% vs. 17.3%, *P* < 0.0001) (Table 2). Although implantation rates were higher in the ionophore group, the difference was not statistically significant. Importantly, ionophore AOA resulted in ongoing pregnancy or delivery in 54.8% of transfer cycles, whereas no ongoing pregnancies occurred in the historical controls (*P* < 0.001). Pregnancy loss was markedly reduced to 12.5% (3/24) in AOA (ionophore-only) cycles compared to history cycles where all pregnancies resulted in missed abortion (3/3).

**Table 2:** Embryology and clinical outcome of couples treated by AOA (ionophore-only) in comparison to their history cycles

Fertilization						
	0%			0–30%		
	History	Ionophore only	*P* value	History	Ionophore only	*P* value
Couples	27			77		
Cycles	31	48		85	133	
Female age	35.7 ± 5	36.5 ± 5		36.2 ± 6	36.8 ± 5	
Male age	38.9 ± 7	39.8 ± 7		38.1 ± 8	38.6 ± 6	
Oocytes retrieved	345	442		991	1556	
MII oocytes injected	277 (80.3)	334 (75.6)	NS	790 (79.7)	1185 (76.2)	NS
Total activated	0	192 (57.5)	< 0.0001	184 (23.2)	739 (62.4)	< 0.0001
2PN (%)	0	168 (50.3)	< 0.0001	137 (17.3)	622 (52.5)	< 0.0001
Cycles with ET	–	16		22	39	
#ET	–	29		24	83	
Implantation	–	8 (27.6)	N/A	3 (12.5)	24 (28.9)	NS
Ongoing/Deliv	–	7 (43.8)	N/A	0 (0)	21 (54.8)	< 0.001
Preg loss	–	1 (12.5)	N/A	3 (100)	3 (12.5)	< 0.001

In a subgroup of patients with a history of complete fertilization failure (*n* = 27), the treatment cycle using ionophore greatly improved fertilization from 0 to 50.3% (*P* < 0.0001) and followed by 43.8% pregnancy rate.

### rhPLCζ-only AOA rescued fertilization deficiency in spermatozoa lacking endogenous PLCζ

Couples treated with rhPLCζ-only AOA targeting sperm PLCζ deficiency demonstrated significant improvements in oocyte activation (47.9% vs. 19.9%, *P* < 0.0001) and fertilization (41.4% vs. 18.3%, *P* < 0.0001) compared with historical cycles (Table 3). Ongoing pregnancy or delivery occurred in 29.4% of rhPLCζ cycles (*P* < 0.05), whereas none were observed in historical cycles. Implantation and pregnancy loss rates showed favorable trends but did not reach statistical significance, likely due to the limited sample size.

**Table 3:** Embryology and clinical outcome of couples treated by AOA (rhPLCζ-only) in comparison to their history cycles

Fertilization						
	0%			0–30%		
	History	rhPLCζ only	*P* value	History	rhPLCζ only	*P* value
Couples	5			20		
Cycles	11	5		40	32	
Female age	37.4 ± 7	38.2 ± 7		36.6 ± 6	37.0 ± 6	
Male age	38.5 ± 6	39.3 ± 6		39.7 ± 8	40.2 ± 8	
Oocytes retrieved	95	53		401	357	
MII oocytes injected	84 (88.4)	44 (83.0)	NS	327 (81.5)	280 (78.4)	NS
Total activated	0	28 (63.6)	< 0.0001	65 (19.9)	134 (47.9)	< 0.0001
2PN (%)	0	21 (47.8)	< 0.0001	60 (18.3)	116 (41.4)	< 0.0001
Cycles with ET	–	3		14	17	
#ET	–	5		23	25	
Implantation	–	1 (20.0)	N/A	2 (8.7)	6 (24.0)	NS
Ongoing/Deliv	–	1 (33.3)	N/A	0 (0)	5 (29.4)	< 0.05
Preg loss	–	0	N/A	2 (100)	1 (16.7)	NS

In five couples with complete fertilization failure due to sperm-related oocyte activation deficiency, AOA by rhPLCζ yielded significantly improved fertilization (0% vs. 47.8%, *P* < 0.0001), with subsequent pregnancy rate of 33.3%.

### Dual AOA targeting borderline, combined, and unexplained etiology of oocyte activation deficiency

The most pronounced improvements were observed with dual AOA in couples with unexplained or combined etiologies (Table 4). Total activation reached 86.8% compared to 27.8% in historical cycles (*P* < 0.0001), and 2PN rates increased threefold (73.2% vs. 24.1%, *P* < 0.0001). Implantation rates increased significantly (31.7% vs. 6.5%, *P* < 0.01), and ongoing pregnancy or delivery occurred in 63.3% of cycles, compared to none in the historical controls (*P* < 0.0001). No pregnancy losses were observed in the combined AOA group.

**Table 4:** Embryology and clinical outcome of couples treated by dual AOA (ionophore + rhPLCζ) in comparison to their history cycles

Fertilization						
	0%			0–30%		
	History	Dual AOA	*P* value	History	Dual AOA	*P* value
Couples	5			38		
Cycles	5	6		48	50	
Female age	36.5 ± 4	37.3 ± 4		36.4 ± 5	37.2 ± 5	
Male age	38.3 ± 6	39.2 ± 6		38.3 ± 7	38.8 ± 7	
Oocytes retrieved	47	60		667	707	
MII oocytes injected	34 (72.3)	45 (75.0)	NS	489 (73.3)	485 (68.6)	NS
Total activated	0	41 (91.1)	< 0.0001	136 (27.8)	421 (86.8)	< 0.0001
2PN (%)	0	31 (68.8)	< 0.0001	118 (24.1)	355 (73.2)	< 0.0001
Cycles with ET	–	5		19	31	
#ET	–	8		31	60	
Implantation	–	2 (25.0)	N/A	2 (6.5)	19 (31.7)	< 0.01
Ongoing/Deliv	–	2 (40.0)	N/A	0 (0)	19 (63.3)	< 0.0001
Preg loss	–	0	N/A	2 (100)	0 (0)	< 0.01

In a subgroup of five patients with history of 0% fertilization, dual AOA boosted fertilization significantly to 68.8% (*P* < 0.0001), granting a subsequent pregnancy of 40.0%.

### Dual AOA normalized fertilization competence of testicular spermatozoa

In a subanalysis of cycles using testicular spermatozoa, dual AOA resulted in striking improvements in activation (92.2% vs. 28.5%, *P* < 0.0001) and fertilization (74.8% vs. 27.1%, *P* < 0.0001) (Table 5). Although differences in implantation and ongoing pregnancy did not reach statistical significance, ongoing pregnancy or delivery was achieved in 63.6% of the combined AOA cycles, whereas none occurred in historical cycles.

**Table 5:** Embryology and clinical outcome of couples utilizing testicular spermatozoa treated by dual AOA (ionophore + rhPLCζ) in comparison to their history cycles

	History	Dual AOA		***P*** value
Couples	11			
Cycles	16		14	
Female age	35.7 ± 4		37.8 ± 4	
Male age	35.5 ± 4		37.6 ± 4	
Oocytes retrieved	181		159	
MII oocytes injected	133 (73.3)		103 (64.8)	NS
Total activated	41 (28.5)		95 (92.2)	< 0.0001
2PN (%)	36 (27.1)		77 (74.8)	< 0.0001
Cycles with ET	3		11	
#ET	6		16	
Implantation	1 (16.7)		7 (43.8)	NS
Ongoing/Deliv	0 (0)		7 (63.6)	NS
Preg loss	1 (100)		0 (0)	NS

### Overall success of AOA in treating patients with recurrent poor ICSI fertilization

A comparison of 215 cycles utilizing AOA with 173 historical non-AOA cycles demonstrated no significant differences in female or male age, number of oocytes retrieved, or proportion of MII oocytes injected (Table 6). However, AOA was associated with a marked improvement in total oocyte activation (66.4% vs. 23.9%, *P* < 0.0001) and hence normal fertilization (2PN: 56.1% vs. 19.6%, *P* < 0.0001).

**Table 6:** Embryology and clinical outcome of couples treated by AOA (all protocols) in comparison to their history cycles

	**History**	**Overall AOA**	***P* value**
Couples	135		
Cycles	173	215	
Female age	36.3 ± 5	36.9 ± 5	
Male age	38.3 ± 7	38.8 ± 7	
Oocytes retrieved	2059	2620	
MII oocytes injected	1606 (78.0)	1950 (74.4)	NS
Total activated	385 (23.9)	1294 (66.4)	< 0.0001
2PN (%)	315 (19.6)	1093 (56.1)	< 0.0001
Cycles with ET	55	87	
#ET	78	168	
Implantation	7 (9.0)	49 (29.2)	< 0.001
Ongoing/Deliv	0 (0)	45 (51.7)	< 0.0001
Preg loss	7 (100)	4 (4.6)	< 0.0001

Clinically, AOA cycles exhibited significantly higher implantation rates (29.2% vs. 9.0%, *P* < 0.001) and resulted in ongoing pregnancy or delivery in more than half of embryo transfer cycles (51.7%), whereas no ongoing pregnancies occurred in historical cycles (*P* < 0.0001). Pregnancy loss was significantly reduced after AOA (4.6% vs. 100%, *P* < 0.0001).

## Discussion

Most fertilization failures have been addressed since the widespread adoption of ICSI. Nonetheless, complete or severe fertilization failure persists in a specific subset of couples even after proper ICSI. AOA using ionomycin or other ionophores has become the most commonly adopted intervention, largely because of its simplicity and ability to induce a rapid intracellular calcium surge [6]. However, clinical experience and previous reports have shown inconsistent benefits of ionophore treatment in overcoming sperm-related activation deficiencies [8, 22], suggesting that an indiscriminate method of inducing a transient calcium surge may not address the underlying biological defects in all cases [21].

To the best of our knowledge, this is the first report on topographic sperm fertilization capability according to sperm origin. Our pilot study results demonstrated that PLCζ content in men with normal spermatogenesis was lowest in testicular spermatozoa than in epididymal and ejaculated counterparts (Fig. 2), explaining the lower fertilization obtained with these immature male gametes [31]. Comparison of PLCζ content across different topographic regions indicates that PLCζ abundance increases post-spermiogenesis, likely due to perinuclear theca remodeling during sperm maturation [36, 37].

In this study, we deliberately challenged the prevailing calcium ionophore approach by investigating gamete-specific contributions to oocyte activation failure. Assessment of PLCζ expression in spermatozoa provided critical mechanistic insight. As presented in Table 1, couples classified as having sperm-related fertilization failure exhibited profoundly reduced PLCζ positivity (5.8 ± 3%), despite otherwise comparable conventional semen parameters, whereas those in the oocyte-related group demonstrated proper PLCζ levels (68.2 ± 17%). These data reinforce the concept that standard semen analysis alone is insufficient to identify sperm-related oocyte activation deficiency and that PLCζ assessment represents a valuable diagnostic tool to tailor treatment [8]. Moreover, the PLCζ assay was corroborated by gene studies carried out on spermatozoa to identify specific mutations related to fertilization.

In patients with a subtle oocyte factor caused by suspected cytoplasmic incompetence, whose spermatozoa retain normal PLCζ level, ionophore-only AOA demonstrated significantly improved activation rate, fully normalized fertilization outcomes, greatly improved implantation, and resulted in successful ongoing pregnancies and deliveries in this subset of patients (Table 2). This suggests that a sudden intracytoplasmic calcium influx may also help address ooplasmic dysmaturity, in contrast to previous findings [20, 22]. Indeed, the calcium ionophore-based AOA reported here yielded similar success to our previous data, which reported comparable fertilization rates [8, 13].

To address sperm-factor oocyte activation deficiency, we demonstrated that the rhPLCζ can be used as a reliable agent to overcome the sperm deficiency. rhPLCζ is a competent protein that can activate oocytes with high efficiency with its safety tested in animal models [38]. Cycles using rhPLCζ-only AOA significantly increased fertilization rates in couples with severe PLCζ deficiency, translating into improved ongoing pregnancies and deliveries compared with historical cycles (Table 3). These results yielded higher ongoing pregnancies than ionophore-only AOA in our previous clinical study (20.9% vs. 29.4%) [8], supporting the conclusion that direct replenishment of deficient PLCζ can support fertilization and sustain embryo development more efficiently than the ionophore-only method.

Notably, in couples with borderline PLCζ levels or unexplained etiologies for their failed fertilization, the dual AOA (ionophore + rhPLCζ) protocol boosted oocyte activation and normal fertilization, resulting in more developing embryos, higher implantation rates, and successful deliveries (Table 4). This combined protocol exceeded the outcomes achieved with either AOA modality alone and suggests a synergistic mechanism: Ionophore exposure may prime the oocyte calcium signaling machinery [20, 39], while rhPLCζ ensures sustained, physiologic-like calcium oscillations [40], simultaneously addressing respective oocyte- and sperm-related fertilizing ability. The reduction in pregnancy loss in this group further supports the notion that appropriate oocyte activation is critical not only for fertilization but also for proper embryo development, while maintaining clinical safety, as confirmed by follow-up of children.

In contrast, cycles using testicular spermatozoa have a lower fertilization threshold [41], given the incomplete maturity of these gametes, than in ejaculated sperms. Dual AOA normalized fertilization rates to levels comparable with standard ICSI using ejaculated spermatozoa. Fertilization increased more than threefold and was associated with encouraging implantation and ongoing delivery rates, with no observed pregnancy loss (Table 5). These findings are clinically relevant, suggesting that dual AOA may partially compensate oocyte activation deficiencies inherent to testicular gametes, a population in which ionophore-only strategies previously yielded limited improvement [42].

From a safety perspective, no adverse effects related to early offspring outcomes have been observed in children assessed by the Ages and Stages Questionnaire, although long-term follow-up remains essential, especially the cycles utilizing rhPLCζ. Our study included detailed sperm–specific gene profiling, enabling systematic PLCζ assessment and a treatment algorithm to guide oocyte activation. The use of a sequence-validated, specific PLCζ represents a methodological strength, providing a targeted and physiological treatment approach. Nevertheless, the study has limitations worth noting. The number of cycles, particularly those using testicular spermatozoa, was relatively limited owing to their low occurrence in ICSI. More broadly, fertilization failure itself occurs in only ~ 1–3% of cases, which inherently constrains the sample sizes of this study [6]. As a result, outcomes such as implantation and ongoing pregnancy should be interpreted with caution and are presented as exploratory. Nonetheless, these findings remain clinically relevant, as they pertain to a rare patient population that has historically been considered difficult or even impossible to treat, thereby providing meaningful insight despite the limited numbers.

In summary, our study provides compelling evidence that individualized AOA strategies guided by sperm PLCζ content can pinpoint gamete-specific deficiencies responsible for fertilization failure and improve both fertilization and clinical outcomes in a targeted manner. The dual ionophore and rhPLCζ approach appears particularly advantageous in cases with mixed or unclear etiology and in cycles using testicular spermatozoa. These findings highlight the novelty and clinical relevance of moving beyond empirical ionophore use toward mechanistic-based AOA, with the potential to redefine standard practice for one of the most challenging problems in assisted reproduction.

## Supplementary Information

Below is the link to the electronic supplementary material.

ESM 1 (124 KB PDF)

## Data Availability

The datasets generated and/or analysed during the current study are available upon request.
