# Supplementary material for: Assisted oocyte activation re-envisioned: a more physiological approach
Source: J Assist Reprod Genet. 2026 Jun 16;43(8):2503–13. doi: 10.1007/s10815-026-03921-2 (PMC13598041; doi:10.1007/s10815-026-03921-2)
Supplement: Supplementary file 1 [file 10815_2026_3921_MOESM1_ESM.pdf]

APPROVED
for use
08-Apr-2025 -
07-Apr-2026

Date (M D Y)	Location Service
Age	Doctor

If No Plate, Print Name, Sex, and History No.

WEILL CORNELL MEDICAL COLLEGE

Consent Form for Clinical Investigation
(If necessary, translate into language of subject)

Project Title: Gamete treatment to correct fertilization failure

Research Project # 0712009553

Principal Investigator: Gianpiero D. Palermo, M.D., Ph.D.

INSTITUTION: Weill Cornell Medical College

INTRODUCTION

You are invited to consider participating in a research study. The study is called "Gamete treatment to correct fertilization failure". You were selected as a possible participant in this study because you are currently undergoing infertility treatment at The Center for Reproductive Medicine (CRM).

Please take your time to make your decision. It is important that you read and understand several general principles that apply to all who take part in our studies:

(a) Taking part in the study is entirely voluntary.

(b) Personal benefit to you may or may not result from taking part in the study, but knowledge gained from your participation may benefit others;

(c) You may decide not to participate in the study or you may decide to stop participating in the study at any time without loss of any benefits to which you are entitled.

The purpose and nature of the study, possible benefits, risks, and discomforts, other options, your rights as a participant, and other information about the study are discussed below. Any new information discovered which might affect your decision to participate or remain in the study will be provided to you. You are urged to ask any questions you have about this study with members of the research team. You should take whatever time you need to discuss the study with your physician and family. The decision to participate or not to participate is yours. If you decide to participate, please sign and date where indicated at the end of this form.

The study will take place only at CRM Embryology and Andrology Laboratories.

WHY IS THE STUDY BEING DONE?

The purpose of this study is to enhance the fertilization rate of couples that seek infertility care. The gametes (egg and sperm) will be treated with different compounds to help the egg become activated and the sperm to undergo capacitation and acrosome reaction, essential steps to obtain fertilization and eventual embryo development. To obtain the highest motile sperm with less DNA damage, the untreated spermatozoa may be obtained by using a microfluidic sperm selection device.

HOW MANY PEOPLE WILL TAKE PART IN THE STUDY?

Participants in the study are referred to as subjects. About 600 (couples) 1200 subjects will be recruited at this site.

WHAT IS INVOLVED IN THE STUDY?

You were selected as a possible participant in this study because you are undergoing infertility treatment with either *in vitro* fertilization (IVF) treatment with intracytoplasmic sperm injection (ICSI) or intrauterine insemination. You may also be selected because you have male factor infertility (including high DNA fragmentation). No additional procedures and tests will be performed on you and your partner besides those that are part of the infertility treatment that have been already discussed with your fertility physician.

The sperm may be selected through a synthetic, sterile, single use microfluidics chip. The spermatozoa will be prepared in the standard fashion and utilized for intra uterine insemination (IUI) or sperm injection (ICSI). If you are undergoing IVF procedures, mature eggs retrieved will be injected with the spermatozoa by the ICSI procedure.

If oocytes are being treated, the spermatozoa may also be prepared in the standard fashion and utilized for injection. The experimental procedure will involve the use of chemicals (streptolysin O that helps in sperm DNA decondensation) that will hopefully help to induce membrane destabilization allowing the sperm to be more efficient in fertilizing the egg. Thereafter, the injected oocytes will be exposed to activating agents (calcium ionophore) to trigger oocyte activation. Sperm with a PLCzeta deficiency will also be treated with a human recombinant PLCzeta protein at the time of ICSI. These eggs will be cultured for 16-18 hours then assessed for the occurrence of fertilization.

Although the successful fertilization of oocytes with absent zona pellucida, the covering that surrounds the oocyte, is still possible with ICSI, the fertilized embryo may fail to develop. If the zona pellucida is missing, an artificial zona pellucida will be created using calcium alginate following ICSI to provide the structural support and protection during embryo development.

The successfully fertilized oocytes will be kept in culture for up to 5 days in the same manner as standard IVF. We will review your pregnancy and delivery results after the study procedure.

HOW LONG WILL I BE IN THE STUDY?

You will be in the study from the time of study procedure till the pregnancy test and till delivery if you become pregnant.

You can stop participating at any time. However, if you decide to stop participating in the study, we encourage you to talk to the researcher and your regular doctor first.

WITHDRAWAL BY INVESTIGATOR, PHYSICIAN, OR SPONSOR

The investigators, physicians or sponsors may stop the study or take you out of the study at any time should they judge that it is in your best interest to do so, if you experience a study-related injury, if you need additional or different medication, or if you do not comply with the study plan. They may remove you from the study for various other administrative and medical reasons. They can do this without your consent.

WHAT ARE THE RISKS OF THE STUDY?

Considering that the study intervention for gamete processing is still new, it may lead to unknown risks: e.g. to the embryo and eventual fetus/baby. Even after gamete processing, there is still a chance that the egg may not fertilize. These techniques are currently not known to pose any additional risk to the sperm or oocytes.

The risks associated with Assisted Reproductive Technologies and pregnancy in general and are not specific to participating in this study. The risks involved with egg retrieval, ICSI, IUI and the utilization of fertility medications will be thoroughly explained to you by your fertility physician and they are all related to the IVF or IUI treatment itself. Your fertility physician will discuss these issues with you in greater detail.

ARE THERE ANY BENEFITS TO TAKING PART IN THE STUDY?

This study may increase your chances of having a fertilized egg and thereby increasing the embryo available for transfer. We cannot and do not guarantee that there will be fertilization after gamete processing. In addition, the information gained from this Study will contribute to the scientific and medical knowledge that may benefit others.

WHAT OTHER OPTIONS ARE THERE?

Instead of being in this study, you have the option to 1) proceed with the standard ICSI or IUI procedure without additional gamete processing or 2) not participate to this study at all.

WHAT ABOUT CONFIDENTIALITY?

Efforts will be made to protect your medical records and other personal information to the extent allowed by law. However, we cannot guarantee absolute confidentiality. Medical records of research study participants are stored and kept according to legal requirements. You will not be identified personally in any reports or publications resulting from this study. Organizations that may request to inspect and/or copy your research and medical records for quality assurance and data analysis include groups such as: Food and Drug Food and Drug Administration, the WCMC, the Institutional Review Board (IRB), and federal research oversight agencies.

If information about your participation in this study is stored in a computer, we will take the following precautions to protect it from unauthorized disclosure, tampering, or damage: subject's identity will be coded and stored in a encrypted computer in a locked office. The computer is under a firewall with the state of the art network where a password is required to log into the system as well as into the file. Only the investigators of this study have access to this data.

HIPAA AUTHORIZATION TO USE or DISCLOSE PROTECTED HEALTH INFORMATION FOR RESEARCH

Purposes for Using or Sharing Protected Health Information: If you decide to join this study, WCMC researchers need your permission to use your protected health information. If you give permission, Weill Cornell Medical College (WCMC) and/or New York-Presbyterian Hospital (NYPH) researchers may use your information or share (disclose) information about you for their research that is considered to be protected health information.

Voluntary Choice: The choice to give WCMC and/or NYPH researcher's permission to use or share your protected health information for their research is voluntary. It is completely up to you. No one can force you to give permission. However, you must give permission for WCMC and/or NYPH researchers to use or share your protected health information if you want to participate in the study. If you decline to sign this form, you cannot participate in this study, because the researchers will not be able to obtain and/or use the information they need in order to conduct their research. Refusing to give permission will not affect your ability to get usual treatment, or health care from WCMC and/or NYPH.

Protected Health Information To Be Used or Shared: Government rules require that researchers get your permission (authorization) to use or share your protected health information. Your medical information may be disclosed to authorize public health or government officials for public health activities when required or authorized by law. If you give permission, the researchers could use or share with the entities identified above any protected health information related to this research study from your medical records and from any test results, which includes **male and female subjects ages, demogragphic, semen analysis, medication information, number of eggs retrieved/injected, fertilization results, and embryo cleavage.**

<u>Other Use and Sharing of Protected Health Information</u>: If you give permission, the researchers could also use your protected health information to develop new procedures or commercial products. They could share your protected health information with the WCMC Institutional Review Board, inspectors who check the research, government agencies and research study staff.

The information that may be shared with the sponsor and/or government agencies could include your medical record and your research record related to this study. They may not be considered covered entities under the Privacy Rule and your information would not be subject to protections under the Privacy Rule.**<u>Confidentiality</u>**: Although the researchers may report their findings in scientific journals or meetings, they will not identify you in their reports. Also, the researchers will try to keep your information confidential, but this cannot be guaranteed. The government does not require everyone who might see your information to keep it confidential, so it might not remain private.

CANCELING AUTHORIZATION

<u>Canceling Permission:</u> If you give the WCMC and/or NYPH researchers permission to use or share your protected health information, you have the right to cancel your permission whenever you want. However, canceling your permission will not apply to information that the researchers have already used or shared.

If you wish to cancel your permission, you may do so at any time by writing to:

Privacy Officer
1300 York Avenue, Box 303
New York, NY 10065

If you have questions about this, call: 646-962-6930 or e-mail: privacy@med.cornell.edu

<u>End of Permission:</u> Unless you cancel it, permission for WCMC and/or NYPH researchers to use or share your protected health information for their research will never end.

ACCESS TO RESEARCH RECORDS

During the course of this study, <u>you will have access</u> to see or copy your protected health information as described in this authorization form in accordance with Weill Cornell Medical College (WCMC) and/or NewYork-Presbyterian Hospital (NYPH) policies. During your participation in this study, you will have access to your research record and any study information that is part of that record.

<u>WHAT ARE THE COSTS?</u>

You or your insurance company will have to pay for the microfluidics procedure and normal IVF and IUI treatment cycle costs. You or your insurance company will be charged for continuing medical care and/or hospitalization that is not a part of the research study.

POLICY/PROCEDURES FOR RESEARCH RELATED INJURY

The Policy and Procedure for Weill Cornell Medical College are as follows:

In accordance with Federal regulations, we are obligated to inform you about the WCMC's policy in the event injury occurs. If, as a result of your participation, you experience injury from known or unknown risks of the research procedures as described, immediate medical care and treatment, including hospitalization, if necessary, will be available at the usual charge for such treatment. No monetary compensation is available from the WCMC or New York Presbyterian Hospital. Further information can be obtained by calling the Committee on Human Rights in Research at (646) 962-8200.

PAYMENT FOR PARTICIPATION

You will not receive payment for participating in this study. You should not expect anyone to pay you for pain, worry, lost income, or other costs that occur from taking part in this research study.

WHAT ARE MY RIGHTS AS A PARTICIPANT?

Taking part in this study is voluntary. You may choose to not take part in the study or to leave the study at any time. If you choose to not participate in the study or to leave the study, your regular care will not be affected nor will your relations with the WCMC, your physicians, or other personnel. In addition, you will not lose any of the benefits to which you are entitled.

WHOM DO I CALL IF I HAVE QUESTIONS OR PROBLEMS?

For questions about the study or a research-related injury, any problems, unexpected physical or psychological discomforts, or if you think that something unusual or unexpected is happening, call Dr. Gianpiero Palermo at 646-962-3689 or Dr. Zev Rosenwaks at 646-962-2764. Be sure to inform the physician of your participation in this study.

If you have questions about your rights as a research participant, contact the WCMC IRB Office. Direct your questions to:

Institutional Review Board at:

Address: 1300 York Avenue
Box 89
New York, New York 10065

Telephone: (646) 962-8200

RESEARCHER'S STATEMENT

I have fully explained this study to the subject. As a representative of this study, I have explained the purpose, the procedures, the benefits and risks that are involved in this research study. Any questions that have been raised have been answered to the individual's satisfaction.

______________________________	______________________________	______________
Signature of person obtaining the consent<br>(Principal Investigator or Co-investigator)	Print Name of Person	Date

SUBJECT'S STATEMENT

I, the undersigned, have been informed about this study's purpose, procedures, possible benefits and risks, and I have received a copy of this consent. I have been given the opportunity to ask questions before I sign, and I have been told that I can ask other questions at any time. I voluntarily agree to participate in this study. I am free to withdraw from the study at any time without need to justify my decision. This withdrawal will not in any way affect my future treatment or medical management and I will not lose any benefits to which I otherwise am entitled. I agree to cooperate with *Dr. Gianpiero D. Palermo* and the research staff and to inform them immediately if *I/we*experience any unexpected or unusual symptoms.

______________________________	______________________________	______________
Signature of Subject	Print Name of Subject	Date

______________________________	______________________________	______________
Signature of Subject	Print Name of Subject	Date
